# Recent Advances in Mitochondria-Targeted Nanoparticle Photodynamic Therapy for Triple-Negative Breast Cancer

**DOI:** 10.3390/ijms27219975

**Published:** 2026-11-07

**Authors:** Agnieszka Przygórzewska, Dorota Bartusik-Aebisher

**Affiliations:** 1 Doctoral School, Medical College of the University of Rzeszów, 35-310 Rzeszów, Poland; agnieszkapr@dokt.ur.edu.pl; 2 Department of Biochemistry and General Chemistry, Medical College of the University of Rzeszów, 35-310 Rzeszów, Poland

**Keywords:** triple-negative breast cancer, TNBC, photodynamic therapy, mitochondria, mitochondrial targeting, nanoparticles, nanoplatforms, photosensitisers, oxidative stress, photoimmunotherapy

## Abstract

Triple-negative breast cancer (TNBC) remains a therapeutically challenging and biologically heterogeneous breast cancer subtype, prompting the development of strategies that exploit specific subcellular vulnerabilities. Mitochondria represent an attractive target for photodynamic therapy (PDT) because the short lifetime and limited diffusion range of photogenerated reactive oxygen species make their site of generation a critical determinant of cellular damage. This review critically examines recent nanoparticle-based approaches to mitochondria-targeted PDT in TNBC, with particular emphasis on nanoplatform design, experimental evidence for mitochondrial localisation, amplification of mitochondrial oxidative stress, regulated cell-death pathways, and preclinical efficacy. A structured search of PubMed, Scopus, and Web of Science identified 3637 records, of which seven original studies ultimately fulfilled the predefined criteria for nanoparticle-mediated classical PDT in TNBC with mitochondrial targeting. The analysed platforms employed intrinsic mitochondrial affinity of photosensitisers, mitochondriotropic ligands, and multistage delivery strategies, while therapeutic amplification involved type I photochemistry, modulation of oxygen availability and consumption, glutathione depletion, metabolic interference, plasmonic enhancement, and combined PDT/PTT. Mitochondrial photodamage was associated with apoptosis, pyroptosis, immunogenic cell death, cGAS–STING signalling, and antitumour immune activation. Despite encouraging preclinical results, the evidence remains limited by the small number of studies, dominance of 4T1-based models, variable strength of mitochondrial-targeting validation, and limited translational assessment. Future development should focus on mechanistically validated targeting, clinically representative TNBC models, simplified nanoplatform design, and demonstration of a clear therapeutic advantage over less complex PDT systems.

## 1. Introduction

Triple-negative breast cancer (TNBC) is a clinically aggressive and biologically heterogeneous subtype of breast cancer, defined by the absence of oestrogen and progesterone receptor expression and the absence of HER2 overexpression or amplification [1]. For many years, the absence of these classic molecular targets limited the options for systemic treatment mainly to chemotherapy. In recent years, however, the therapeutic landscape for TNBC has expanded significantly thanks to the use of immune checkpoint inhibitors, PARP inhibitors in appropriately selected patients, and antibody–drug conjugates [2,3]. Despite this, treatment resistance, relapses and metastatic progression remain significant clinical problems [4]. Furthermore, TNBC is not a single, biologically homogeneous entity, but encompasses tumours that differ in their molecular, immunological and metabolic profiles, which justifies the search for strategies that exploit specific vulnerabilities of cancer cells [5,6].

Photodynamic therapy (PDT) is based on the activation of a photosensitiser (PS) by light of a specific wavelength, leading to the formation of reactive oxygen species (ROS) and localised cell damage [7,8]. The ability to control the cytotoxic effect spatially and temporally through the positioning of photosensitisers and the local delivery of light is one of the main advantages of PDT. However, its effectiveness is limited by, amongst other things, the poor solubility and insufficient selectivity of many photosensitisers, tumour hypoxia, limited light penetration and the uncontrolled distribution of PS following cellular internalisation [9,10]. The use of nanocarriers can partially overcome these barriers by improving the solubility and stability of PS, enhancing its delivery to the tumour and cellular uptake, controlling its release, and enabling the integration of PDT with additional therapeutic mechanisms [8].

However, the efficacy of PDT depends not only on the total amount of photosensitiser delivered to the cell, but also on its subcellular localisation [11]. Photogenerated ROS are characterised by a short lifetime and a limited diffusion range; consequently, the initial oxidative damage occurs primarily in the immediate vicinity of the site where they are generated [12]. Mitochondria are therefore a particularly attractive target for PDT, as they play a role in the regulation of energy metabolism, redox homeostasis and programmed cell death. In TNBC, mitochondrial dysfunction and metabolic reprogramming are further linked to tumour progression, adaptation to stress and the development of therapeutic resistance. Importantly, mitochondrial dependence is unlikely to be uniform across TNBC, as different molecular and metabolic phenotypes may vary in their reliance on glycolysis, oxidative phosphorylation and other mitochondrial functions. Consequently, susceptibility to mitochondria-targeted PDT may also differ between tumours, and mitochondrial targeting should not be assumed to provide an equivalent therapeutic benefit across all TNBC subtypes. Targeting ROS photogenesis at mitochondria may therefore enhance the biological consequences of short-term oxidative stress by directly disrupting bioenergetics, redox balance and the mitochondrial mechanisms that control cell survival [13].

The mitochondrial localisation of a photosensitiser may result from its inherent physicochemical properties, the use of mitochondriotropic targeting moieties, or multi-stage delivery systems that combine selectivity at the tumour or cellular level with subsequent subcellular targeting [14,15]. Nanoplatforms also make it possible to enhance mitochondrial oxidative stress by modifying the type of ROS generated, regulating oxygen availability and consumption, impairing antioxidant systems, disrupting mitochondrial metabolism, or combining PDT with other therapeutic modalities [16,17]. However, a distinction must be made between the accumulation of the nanoplatform within the tumour, its internalisation by the cell, the mitochondrial localisation of the released photosensitiser, and the actual delivery of an intact nanoparticle to the mitochondrion, as these processes are not mechanistically equivalent.

The aim of this review is to provide a critical analysis of the latest nanoplatforms used for mitochondrially targeted PDT in TNBC, with particular emphasis on experimental evidence confirming mitochondrial localisation and the functional distinction between the roles of the nanocarrier and the photosensitiser. Strategies for enhancing mitochondrial oxidative stress, the consequences of mitochondrial photodamage—including regulated mechanisms of cell death and the immune response—and current preclinical evidence are discussed. Particular attention is also paid to methodological and translational limitations, as well as to the question of whether mitochondrial targeting provides a genuine therapeutic advantage over less complex PDT systems.

## 2. Literature Search and Study Selection

A structured literature search was conducted in PubMed, Scopus, and Web of Science to identify original experimental studies relevant to nanoparticle-mediated photodynamic therapy in breast cancer. The same Boolean query, “PDT AND nanoparticles”, was applied in all three databases without database-specific modification. The search covered studies published between January 2025 and August 2026.

Studies were considered eligible if they reported original experimental research involving nanoparticle-based classical photodynamic therapy in breast cancer. Review articles and other non-original publications, studies unrelated to breast cancer, and studies lacking either a nanoparticle-based delivery system or a classical photodynamic component were excluded. Following this initial screening, studies were further assessed for relevance to triple-negative breast cancer and evidence of mitochondrial targeting.

A total of 3637 records were initially identified. After removal of 1848 duplicates, 1789 records remained for screening. Of these, 357 non-original publications were excluded, leaving 1432 original studies. A further 1146 studies unrelated to breast cancer were excluded, resulting in 286 breast cancer studies. Twenty-one studies did not meet the combined criterion of nanoparticle-based delivery and classical PDT, leaving 265 original studies on nanoparticle-mediated PDT in breast cancer. Application of the final eligibility criteria requiring both TNBC relevance and mitochondrial targeting excluded 258 additional studies, resulting in seven original articles included in the qualitative synthesis.

Potential publication and indexing bias should be considered when interpreting this evidence base. Studies reporting strong therapeutic effects or successful mitochondrial targeting may be more likely to be published than studies showing limited efficacy, negative findings, or unsuccessful targeting. In addition, the use of the specific search terms “PDT” and “nanoparticles” may have failed to identify relevant studies employing alternative terminology in their titles, abstracts, keywords, or database indexing. Therefore, the seven included studies should be regarded as the identifiable published evidence meeting the predefined criteria rather than as a complete representation of all experimental work performed in this field. Moreover, because only seven studies met the final eligibility criteria and these differed substantially in photosensitiser chemistry, nanoplatform architecture, mitochondrial-targeting strategy, irradiation parameters, biological models and outcome measures, the present review should be regarded as a qualitative and hypothesis-generating synthesis rather than as evidence of an established therapeutic advantage of mitochondria-targeted nanoparticle PDT in TNBC.

## 3. Design of Nanoplatforms and Strategies for Targeting Mitochondria

**Figure 1:**
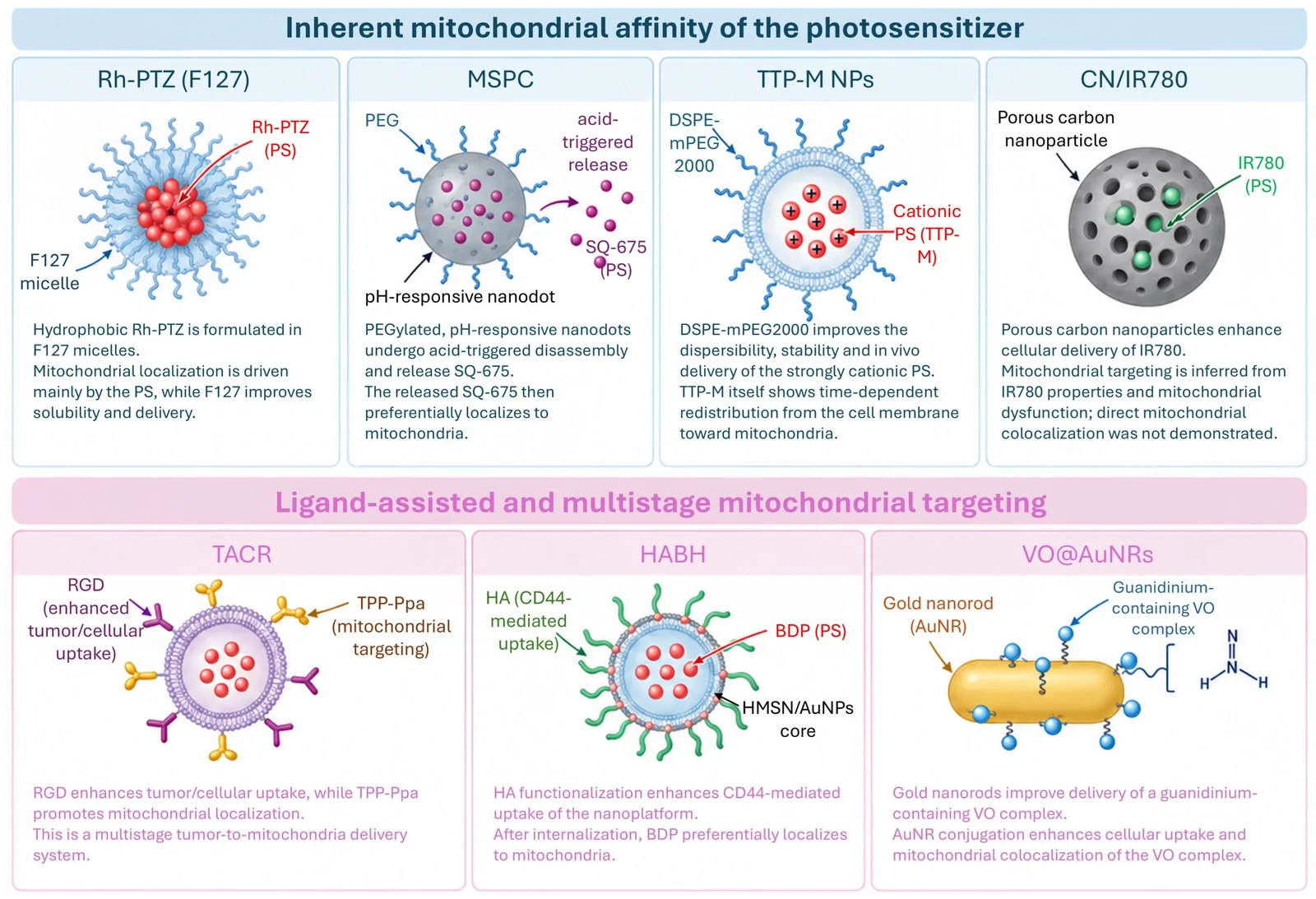
Representative nanoplatforms and structural strategies for mitochondrial targeting in nanoparticle-mediated photodynamic therapy of triple-negative breast cancer.

The efficacy of mitochondria-targeted photodynamic therapy depends not only on the effective uptake of the photosensitiser (PS) by cancer cells, but also on its subcellular distribution, and in particular on its ability to accumulate in the mitochondria [11]. This is of particular significance given the short lifetime and limited diffusion range of photogenerated reactive oxygen species, particularly singlet oxygen (1O_2_), which means that primary photodynamic damage occurs mainly in the vicinity of the site of its generation [18]. For this reason, nanoplatforms developed for the treatment of TNBC employ various strategies designed to increase mitochondrial exposure to the component responsible for the photodynamic effect. However, a clear distinction must be made between mitochondrial targeting and the accumulation of the nanoplatform within the tumour, its internalisation by cancer cells, and the secondary damage to mitochondria following PDT. In many systems, the nanocarrier primarily serves to improve the solubility and stability of the photosensitiser, its delivery to the tumour and cellular uptake, or to control the release of the payload; however, the final mitochondrial localisation may result from the physicochemical properties of the PS itself or from the presence of a targeting motif attached to the PS or the nanocarrier [19]. Therefore, the term ‘mitochondria-targeted nanoparticle PDT’ may, in practice, refer to nanoparticle-mediated delivery of a photosensitiser that exhibits an affinity for mitochondria, rather than unequivocally confirmed penetration of the entire nanostructure into the mitochondrion. Throughout this review, the term “mitochondrial localisation” refers to the experimentally observed localisation of the photosensitiser or other fluorescent cargo unless the nanocarrier itself was independently tracked. The terms “mitochondrial delivery” or “mitochondrial internalisation of the nanoplatform” are reserved for situations in which the presence of the intact carrier within mitochondria has been directly demonstrated (Figure 1).

### 3.1. Mitochondrial Affinity Resulting from the Structure of the Photosensitiser

One of the key strategies is the use of photosensitisers, whose inherent physicochemical properties—such as charge, lipophilicity and ability to interact with membranes, favour their preferential localisation in mitochondria, whilst the nanoparticle primarily acts as a carrier to improve their solubility, stability, delivery or cellular uptake [20,21]. This approach avoids the need for additional functionalisation of the nanocarrier surface with a mitochondrial ligand and has been utilised in several recent systems designed for the treatment of TNBC. Duan et al. developed the photosensitiser Rh-PTZ, which, due to its hydrophobic nature, was encapsulated in the amphiphilic copolymer Pluronic F127. In this system, mitochondrial affinity stemmed primarily from the properties of Rh-PTZ itself, whilst F127 acted as a carrier to facilitate its delivery. In 4T1 cells, Rh-PTZ fluorescence showed strong colocalisation with MitoTracker Green (PCC = 0.91) and significantly weaker colocalisation with a lysosomal marker, indicating preferential localisation of the photosensitiser in the mitochondria. It should be emphasised, however, that the colocalisation analysis was based on Rh-PTZ fluorescence and therefore confirms the mitochondrial localisation of PS, but does not directly demonstrate the presence of whole F127 nanoparticles in the mitochondria [22]. Yao et al. employed a similar strategy, using the mitochondriotropic photosensitiser SQ-675 encapsulated in acid-sensitive nanodots derived from MOF structures (MSPC). In this case, the nanocarrier degraded in the acidic intracellular environment, allowing the release of SQ-675, which then localised preferentially in the mitochondria. In 4T1 cells, the colocalisation of the SQ-675 signal with MitoTracker increased over time, with the PCC rising from 0.58 after 2 h to 0.82 after 4 h and approximately 0.84–0.88 after 6 h. Free SQ-675 also exhibited mitochondrial affinity, indicating that the final subcellular localisation resulted primarily from the properties of the photosensitiser itself, whilst the nanocarrier mainly served to control its delivery and release [23]. These data therefore support a two-stage model in which the nanoplatform facilitates the intracellular release of PS, whilst its own properties determine its subsequent mitochondrial localisation. Pu et al. utilised the dicationic photosensitiser TTP-M, whose properties facilitated time-dependent subcellular redistribution. Initially, TTP-M localised mainly within the cell membrane; however, during further incubation, it migrated to the mitochondria, achieving high colocalisation with a mitochondrial marker (PCC = 0.95) in HeLa cells. Encapsulation of TTP-M in DSPE-mPEG2000 improved the formulation’s properties and masked the free molecule’s strongly positive charge without compromising its ability to subsequently localise to the mitochondria; after 120 min, the reported colocalisation coefficient was 0.9687. These results indicate that TTP-M itself was primarily responsible for the final subcellular localisation, whilst the nanocarrier facilitated its delivery. It should be noted, however, that the detailed analysis of mitochondrial colocalisation was carried out mainly in HeLa cells, whilst the system’s anticancer activity in the context of TNBC was assessed primarily in 4T1 models. Consequently, the mitochondrial localisation mechanism has not been validated with comparable detail directly in TNBC models [24]. Another example of a photosensitiser exhibiting an inherent tendency to accumulate in mitochondria is IR780, a lipophilic cationic heptamethylcyanine dye. It has been shown to accumulate preferentially in cancer cells and to localise predominantly in the mitochondria [25]. However, the IR780 uptake mechanism is more complex than a simple process dependent on mitochondrial membrane potential; mechanistic studies indicate a significant role for energy-dependent transport and OATP transporters, in particular OATP1B3 [26]. Gu et al. incorporated IR780 into hollow, porous nitrogen-doped carbon nanoparticles derived from polydopamine and subsequently modified with DSPE-PEG, creating CN/IR780 for combined PDT and photothermal therapy. The nanocarrier significantly enhanced the delivery of IR780 to 4T1 cells, and the therapy administered led to a marked reduction in mitochondrial membrane potential. However, the study did not include a direct analysis of mitochondrial colocalisation using an organelle marker. The mitochondrial targeting of CN/IR780 was therefore inferred mainly on the basis of the known properties of IR780 and the subsequently observed mitochondrial dysfunction. Since mitochondrial membrane depolarisation is a consequence of photodynamic damage, rather than direct evidence of prior PS localisation within the mitochondria, CN/IR780 should be regarded as a system with a biologically plausible, yet less directly documented, affinity for mitochondria than Rh-PTZ, SQ-675 or the systems described below [27].

### 3.2. Ligand-Dependent Mitochondrial Targeting

The second main strategy involves the covalent introduction of positively charged groups with mitochondrial affinity. One of the best-studied of these is the triphenylphosphonium cation (TPP). Its delocalised positive charge and lipophilic nature facilitate penetration through biological membranes, whilst the strongly negative electrical potential on the inner side of the inner mitochondrial membrane drives the accumulation of TPP conjugates in the mitochondrial matrix [28,29]. TPP primarily ensures subcellular targeting towards the mitochondria, rather than specific recognition of cancer cells. Therefore, in anti-cancer systems, its action can be combined with separate mechanisms that enhance the selectivity of delivery to the tumour or cancer cells [30]. This strategy was applied by Wang et al. in the TACR system, in which pyropheophorbide a (Ppa) was modified with a TPP group and subsequently integrated with atowakwon, catalase and RGD-PEG-BSA. An analogous PACR system containing Ppa without TPP was prepared as a control, enabling a direct assessment of the effect of this group on subcellular localisation. In 4T1 cells, TACR showed greater colocalisation with MitoTracker than PACR, with PCC values after 4 h of 0.54 and 0.36, respectively. This result supports the role of TPP in enhancing the mitochondrial localisation of the photosensitiser, although the moderate PCC value indicates that the distribution of TACR was not exclusively mitochondrial. As the analysed signal originated from PS, these data primarily confirm the increased localisation of TPP-Ppa within the mitochondria, rather than directly indicating the presence of the entire multi-component TACR nanoplatform within this organelle [31]. Guanidinium motifs represent an alternative strategy. Guanidinium groups remain positively charged under physiological conditions and can form strong electrostatic interactions and multipoint hydrogen bonds with the anionic components of membranes, which promotes their association with the membrane and facilitates cellular internalisation [32]. Unlike TPP, the action of guanidinium motifs is not based on the classical lipophilic cation mechanism. Their ability to direct compounds to the mitochondria also depends on the properties of the entire molecular structure. It has been shown, amongst other things, that a change in the length of the linkers and the associated lipophilicity can significantly alter the preferential localisation of guanidinium-rich transporters between the mitochondria and endolysosomal compartments [33,34]. This strategy was utilised by Bag et al., who conjugated an oxovanadium(IV) complex containing a guanidinium group with plasmonic gold nanorods, yielding VO@AuNRs. In MDA-MB-231 cells, both the free VO complex and VO@AuNRs exhibited preferential mitochondrial localisation; however, the PCC with MitoTracker increased from 0.63 for VO to 0.94 for VO@AuNRs. This result indicates that conjugation with nanorods significantly enhanced the mitochondrial colocalisation of the complex. The authors proposed that the high density of complexes containing guanidinium groups on the surface of the nanorods promotes cooperative electrostatic interactions with the anionic components of the membranes and enhanced cellular uptake [35]. The increased mitochondrial colocalisation of VO@AuNRs may therefore reflect both greater internalisation of the system and the mitochondrial affinity of the VO complex itself. These mechanisms should not be conflated when assessing actual organelle-specificity.

### 3.3. Integration of Cellular and Mitochondrial Targeting in Multi-Stage Delivery Systems

Since the presence of a mitochondriotrophic motif does not in itself ensure selective delivery to tumour tissue, hierarchical systems combine a mechanism that enhances delivery to the tumour or uptake by tumour cells with a separate mechanism that directs the cargo to the mitochondria [36,37]. This approach is particularly useful in TNBC, as it allows for the functional separation of two levels of targeting: the component responsible for tumour recognition or increased cellular uptake acts in the first stage, whilst the photosensitiser or additional mitochondriotropic ligand determines the preferential subcellular localisation following internalisation or release [13,38]. One example of a hierarchical structure is the HABH nanoplatform developed by Xu et al., consisting of gold nanoparticles deposited on hollow mesoporous silica nanoparticles (HMSN/AuNPs), containing the type I BODIPY photosensitiser (BDP) and coated with hyaluronic acid (HA) [39]. In this system, HA acted as the first-level targeting agent via its affinity for the CD44 receptor. The HA–CD44 axis is of particular interest in TNBC, as CD44 is frequently overexpressed in cells of this subtype, including populations with tumour stem cell characteristics, and is involved, amongst other things, in adhesion, migration, EMT and tumour progression [40]. However, HA does not provide absolute specificity for TNBC, as CD44 is also present in normal cells; its use is intended rather to enhance the preferential binding and receptor-mediated internalisation of the nanocarrier by cells with high CD44 expression [41]. The authors assessed the significance of this mechanism in 4T1 cells, which were pre-incubated for 2 h with increasing concentrations of free HA prior to exposure to HABH. The reduction in HABH uptake following such competitive inhibition supports the involvement of interactions between HA and its surface receptors in the internalisation process. Upon entering the cell, a second stage of targeting took place: the nanocarrier delivered and released the mitochondriotrophic BDP, which then redistributed towards the mitochondria. The authors assessed this localisation independently of cellular uptake itself, by comparing the BDP signal with markers for mitochondria (Rhodamine 123), lysosomes, the Golgi apparatus, the endoplasmic reticulum and the nucleus; the greatest overlap in signals was observed for mitochondria [39]. In this case, it is therefore important to distinguish between two distinct processes: HA increases the delivery of HABH to cancer cells via a mechanism involving CD44, whilst, following internalisation, it is the released BDP—rather than HA or the entire HMSN/AuNPs structure—that is responsible for preferential mitochondrial localisation [39]. BDP is a derivative of boron-dipyrromethen (BODIPY) designed as a type I photosensitiser. Its structure comprises an extensive π-conjugated system and a cationic indole moiety, which confers on the molecule properties consistent with the typical mechanism of mitochondrial accumulation of lipophilic cations. Compounds of this type can penetrate biological membranes, and the negative electrical potential of the inner mitochondrial membrane promotes their accumulation within the mitochondria [42]. In the case of BDP, its preferential mitochondrial localisation was directly assessed in 4T1 cells following a 4 h incubation with HABH through a parallel analysis of the colocalisation of the BDP signal with markers of the mitochondria, the Golgi apparatus, lysosomes, the endoplasmic reticulum and the cell nucleus. The highest colocalisation was observed for mitochondria labelled with Rhodamine 123 (PCC = 0.92; OLC = 0.93). By comparison, the PCC/OLC values were 0.76/0.79 for the endoplasmic reticulum, 0.41/0.45 for the Golgi apparatus, 0.36/0.44 for lysosomes and 0.28/0.39 for the cell nucleus [39]. These data therefore indicate a clearly preferential, though not exclusive, localisation of BDP within the mitochondria. The significant colocalisation with the endoplasmic reticulum suggests that it is more accurate to describe BDP as a photosensitiser with a preferential affinity for mitochondria rather than as a ‘mitochondria-specific’ compound. A similar principle of hierarchical targeting was applied by Wang et al. in the TACR nanoplatform; however, in this case, the two stages of targeting were assigned to two separate functional modules. The first of these was RGD-PEG-BSA, in which the RGDfC peptide acted as a ligand directing the nanoparticle towards cancer cells [31]. The RGDfC peptide, corresponding to the cyclic pentapeptide c(RGDfC) [cyclo(Arg-Gly-Asp-D-Phe-Cys)], exhibits high affinity for the αvβ3 integrin. However, its binding is not entirely restricted to αvβ3, as c(RGDfC) can also interact with other RGD-binding integrins, including αvβ5 and α5β1 [43]. The αvβ3 integrin is a particularly interesting target in TNBC, as its increased expression has been reported on the surface of cells of this breast cancer subtype; this receptor is also present on the activated endothelium of tumour vessels, which allows RGD to potentially interact with both the tumour cells and its vasculature [44,45]. However, αvβ3 is not specific to tumour tissue. It occurs physiologically, amongst other places, on osteoclasts, and its expression is also observed in activated macrophages, migrating smooth muscle cells and angiogenic endothelial cells; its expression may also be increased during inflammatory processes and tissue repair [46]. Therefore, RGD modification should be interpreted as a mechanism that enhances preferential delivery to cells and tissues with high levels of RGD-dependent integrins, rather than as a strictly specific targeting of TNBC. The significance of RGD in TACR was assessed by comparison with the analogous TACP system, in which RGD-PEG-BSA was replaced with mPEG-BSA. After 2 and 4 h of incubation with 4T1 cells, TACR exhibited a stronger intracellular signal than the formulation lacking RGD, both in flow cytometry and confocal imaging, which supports the contribution of the RGD modification to increased cellular uptake. The authors also observed greater accumulation of the RGD-containing formulation in 4T1 tumours in vivo than in the corresponding formulation without RGD. It should be noted, however, that this study did not directly measure αvβ3 expression in 4T1 cells, nor did it conduct a blocking experiment using free RGD or an anti-αvβ3 antibody. These data therefore confirm the importance of RGD presence for increased uptake, but do not allow the entire effect to be unequivocally attributed solely to an αvβ3-dependent mechanism [31]. The second stage of hierarchical targeting was carried out by the mitochondriotrophic TPP-Ppa, whose mechanism of action and the effect of TPP on mitochondrial localisation have been discussed above. In the context of TACR, the experimental design itself is therefore particularly important: the use of a TACP formulation lacking RGD and a PACR formulation lacking TPP enabled an independent assessment of two successive stages of delivery—increased cellular uptake dependent on RGD modification and subsequent enrichment of the photosensitiser in the mitochondria dependent on TPP [31]. This distinction is methodologically significant, as the increased mitochondrial localisation observed for the nanoplatform may be partly due to an increase in the total amount of photosensitiser delivered to the cell, rather than solely to an improvement in its subcellular selectivity. Hierarchical delivery to the tumour and mitochondria can also be achieved without the use of two separate receptor ligands. In such systems, the first level of selectivity stems rather from the physicochemical properties of the nanocarrier or its response to the tumour microenvironment, whilst the second is related to the inherent affinity of the released photosensitiser for the mitochondria [36,37]. An example of using the tumour microenvironment as the first level of selectivity is the MSPC system developed by Yao et al. [23]. This strategy exploits the tendency of many TNBC tumours to acidify their microenvironment. TNBC often exhibits enhanced glycolytic metabolism, accompanied by increased glucose consumption, lactate production, and enhanced export of lactate and protons via monocarboxylate transporters. These processes lead to a reduction in the pH of the tumour’s extracellular space relative to normal tissues [47]. In 4T1 models, acidification of the tumour microenvironment has also been demonstrated in vivo using pH imaging techniques, which further justifies the use of this stimulus in systems designed for TNBC [48].

The MSPC was constructed from ultra-small MOF nanodots containing SQ-675, PEITC and CORM-401, as well as modified DSPE-PEG2000. The system did not contain a ligand that recognises a specific TNBC cell receptor; instead, it exploited the difference between the relatively neutral conditions of the circulation and the more acidic tumour microenvironment. The MOF structure was stable at pH 7.4, but became increasingly destabilised as the pH decreased. At pH 6.0, used by the authors as a model for an acidic TME, PEITC release increased to approximately 91.3% within 60 min, whilst at pH 7.4 it remained below 10%. The breakdown of the nanocarrier also led to the release of the poorly soluble SQ-675, which subsequently aggregated, increasing the hydrodynamic size of the structures to approximately 330 nm. It should be noted, however, that the pH of 6.0 used in the in vitro experiments [23] is lower than the average extracellular pH of most solid tumours, which is usually around 6.2–6.9 [49]. This means that significant destabilisation of MSPC is likely to occur primarily in the most acidic regions of the tumour and following internalisation into acidic endosomal-lysosomal compartments. The pH-responsiveness of MSPC should therefore be regarded as a mechanism that enhances preferential release and retention in the tumour microenvironment, rather than as a specific recognition of TNBC cells.

### 3.4. The Importance of Nanocarrier Architecture for Intracellular Delivery and Mitochondrial Targeting

The architecture of the nanocarrier influences the efficacy of mitochondrial PDT primarily by improving the solubility and stability of the photosensitiser, increasing its cellular uptake and controlling its intracellular release [36]. In polymer systems such as F127-Rh-PTZ [22] and TTP-M/DSPE-mPEG2000 [24], The carrier primarily serves a formulation function, whilst mitochondrial affinity remains a property of the photosensitiser itself. In contrast, degradable and porous nanostructures, such as MSPC [23] and HABH [39], enable the controlled release of the payload and the integration of additional therapeutic functions. In more complex systems, such as TACR [31], VO@AuNRs [35] or CN/IR780 [27], A nanocarrier may simultaneously enhance cellular uptake, modify photophysical or photothermal properties, or contribute to the regulation of therapeutic conditions. In most of the platforms analysed, however, the nanocarrier does not act as a direct mitochondrial-targeting element; its primary role is to enable the effective delivery of a photosensitiser, which subsequently exhibits preferential subcellular localisation. Significant differences, however, relate to the strength of the experimental evidence for mitochondrial targeting. Direct quantitative colocalisation in TNBC models was demonstrated for Rh-PTZ in 4T1 cells, where the PCC was 0.91 [22], for BDP supplied by HABH in 4T1 cells, where the PCC was 0.92 [39], and for MSPC/SQ-675 in 4T1 cells, where PCC values increased over time to approximately 0.84–0.88 [23]. In the case of TACR in 4T1 cells, the PCC was 0.54, whilst for the corresponding system lacking TPP it was 0.36 [31]. VO@AuNRs in MDA-MB-231 cells, on the other hand, exhibited a PCC of 0.94, compared with 0.63 for the free VO complex [35]. TTP-M also showed mitochondrial colocalisation; however, the most detailed analyses were carried out mainly in HeLa cells, rather than in TNBC cells [24]. In the case of CN/IR780, mitochondrial targeting was inferred indirectly on the basis of the properties of IR780 and the depolarisation of the mitochondrial membrane observed following treatment, without direct examination of colocalisation with a mitochondrial marker [50]. It should be emphasised that fluorescence colocalisation confirms the spatial co-occurrence of the photosensitiser signal with the mitochondrial marker; however, it does not in itself prove that the entire nanoparticle is transported into the mitochondrion, nor does it allow for an unambiguous determination of its submitochondrial localisation [51,52]. This limitation is particularly significant in systems where the recorded signal originates from the photosensitiser itself or from a single fluorescent label on a nanocarrier, as the release or redistribution of the fluorophore may lead to a discrepancy between the location of the signal and the location of the intact nanoparticle [53,54]. Accordingly, PCC values should be interpreted as quantitative measures of spatial correlation between two fluorescence signals rather than as direct evidence of mitochondrial internalisation of an intact nanoplatform. Even a high PCC may reflect mitochondrial accumulation of a released or redistributed photosensitiser while the nanocarrier itself remains outside the organelle; therefore, PCC should be interpreted together with information on the origin of the detected fluorescence signal and, where possible, complementary biochemical or high-resolution imaging approaches. Fluorescence colocalisation is also sensitive to methodological factors unrelated to true organelle targeting. PCC values may be influenced by image acquisition conditions, background signal, thresholding and selection of the analysed region, while the diffraction-limited resolution of conventional fluorescence microscopy does not establish physical entry of a fluorescent species into the mitochondrial compartment [52]. In nanoparticle systems, interpretation is further complicated by fluorophore dissociation or dye leakage, because the detected fluorescence may no longer accurately represent the location of the intact nanocarrier [53,54]. Consequently, differences in PCC obtained across separate studies using different imaging protocols should not be interpreted as direct quantitative differences in mitochondrial-targeting efficiency. In future studies, it would therefore be advisable to supplement colocalisation analyses with complementary methods, such as the isolation of the mitochondrial fraction and the direct quantification of the target cargo or nanocarrier component within the mitochondria, as well as higher-resolution imaging techniques [51]. In the case of systems where the targeting mechanism is thought to depend on the mitochondrial membrane potential, experiments involving depolarisation of ΔΨm—for example, using FCCP—provide additional mechanistic insight. This is particularly relevant for TPP conjugates, as their mitochondrial accumulation is dependent on the membrane potential, and the abolition of ΔΨm using FCCP significantly reduces their mitochondrial uptake [28,29]. Taken together, the available data suggest that the efficacy of mitochondrial PDT in TNBC often depends on a functional division between the carrier and the photosensitiser: the nanoplatform primarily facilitates payload delivery and controls its availability, whereas the molecular properties of the released photosensitiser or a dedicated mitochondriotropic ligand determine its subsequent mitochondrial localisation.

## 4. Strategies for Enhancing Mitochondrial Oxidative Stress

Simply delivering the photosensitiser to the mitochondria does not guarantee high PDT efficacy. The final outcome also depends on the nature of the photochemical reactions taking place and the type of ROS generated [55], the availability of oxygen at the reaction site [56], the ability of cells to neutralise oxidative stress via antioxidant systems [57], and the activation of adaptive response pathways to oxidative stress [58]. The nanoplatforms under investigation employ several complementary strategies to enhance mitochondrial oxidative stress: shifting photochemistry towards Type I reactions, increasing the availability of oxygen or limiting its consumption, impairing antioxidant systems and energy metabolism, and improving the spatial and photophysical utilisation of the ROS generated.

### 4.1. Type I PDT as a Strategy to Mitigate the Effects of Hypoxia

In comparison with the type II mechanism, which is based on the transfer of energy to molecular oxygen and leads mainly to the formation of singlet oxygen (^1^O_2_), the Type I mechanism involves the transfer of an electron or a hydrogen atom between an excited photosensitiser and the surrounding substrates, leading to the formation of radical ROS, such as O_2_•^−^, and subsequently, amongst others, H_2_O_2_ and •OH. As Type I reactions require less oxygen than the classical Type II mechanism, they may retain some efficacy under hypoxic conditions and are therefore being developed as a strategy to mitigate the impact of tumour hypoxia on PDT [59,60,61]. A clear example is Rh-PTZ, which, upon photoactivation, primarily generated the superoxide anion radical (O_2_•−) and the hydroxyl radical (•OH), whilst the production of ^1O_2_ was minimal; after 60 s of irradiation, the decrease in the absorbance of the ABDA probe was only 0.18 per cent. The generation of O_2_•− and •OH persisted even under hypoxic conditions, as confirmed by both specific fluorescent probes and ESR measurements. In 4T1 cells, irradiation with Rh-PTZ led to intense production of mitochondrial ROS and depolarisation of the mitochondrial membrane. Importantly, cytotoxic efficacy was largely maintained under conditions of limited oxygen availability: following PDT, the viability of 4T1 cells was <18 per cent under normoxia and <24 per cent under hypoxia [22]. These results suggest that the combination of type I photochemistry with mitochondrial localisation may partially mitigate the adverse effects of hypoxia on PDT. A similar strategy was employed in HABH, where the mitochondriotropic BODIPY BDP primarily generated •OH via a Type I mechanism. The formation of •OH was confirmed by ESR, and the photodynamic activity of BDP was maintained under various atmospheric conditions. However, the HABH system enhanced oxidative stress via an additional mechanism: Au nanoparticles deposited on HMSN exhibited activity similar to that of glucose oxidase and peroxidase. The first stage led to the formation of H_2_O_2_ using glucose, whilst the peroxidase-like activity enabled the further conversion of H_2_O_2_ to •OH. In 4T1 cells, the combination of HABH with irradiation induced the highest ROS signal and a marked loss of mitochondrial membrane potential. However, the HABH mechanism requires a significant caveat. Although the authors describe BDP-mediated type I PDT as oxygen-independent, the activity of the GOx-like component of HMSN/AuNPs was markedly reduced in a N_2_ atmosphere and increased in the presence of O_2_ [39]. The entire nanoplatform should not, therefore, be regarded as a completely oxygen-independent system. It is more accurate to say that HABH combines Type I photochemistry, which is less dependent on oxygen, with an additional catalytic cascade that increases the production of •OH.

### 4.2. Increasing Oxygen Availability and Reducing Its Mitochondrial Consumption

An alternative to altering the photochemical mechanism is to directly modify the cell’s oxygen balance. This strategy was employed in TACR, in which catalase (CAT) and atovaquone (ATO) perform complementary functions [31]. A key element of this strategy is the exploitation of the disrupted redox homeostasis of the TME, in which an increased availability of H_2_O_2_ is observed in many tumours compared with normal tissues [62]. Endogenous H_2_O_2_ can serve as a local substrate for the enzymatic production of oxygen, a property that has been utilised in nanoplatforms containing catalase (CAT). Encapsulated CAT retains the ability to catalytically decompose H_2_O_2_ into H_2_O and O_2_, increasing the local availability of oxygen and partially alleviating hypoxia, thereby improving conditions for oxygen-dependent PDT [63]. A complementary mechanism is provided by atovaquone (ATO), an inhibitor of the mitochondrial electron transport chain that acts primarily on complex III; inhibition of this complex reduces mitochondrial oxygen consumption [64]. The combination of these two mechanisms therefore makes it possible to simultaneously increase the local supply of O_2_ through the conversion of H_2_O_2_ and to reduce its consumption by mitochondrial respiration, thereby increasing the pool of oxygen available for the photodynamic reaction [31]. The Seahorse analysis revealed a dose-dependent inhibition of mitochondrial oxygen consumption in 4T1 cells following exposure to TACR; at 20 μg/mL, the lowest OCR value was observed, along with a reduction in maximum respiration and ATP production. At the same time, the combination of both oxygen regulatory mechanisms increased intracellular ROS production compared with systems lacking CAT or ATO. Under hypoxic conditions, the viability of 4T1 cells following TACR + light fell to approximately 8% at a concentration of 10 μg/mL, whilst for systems containing only one of the two hypoxia-regulating components, it remained at around 30%. In vivo, too, TACR more strongly attenuated the hypoxia probe signal than formulations lacking ATO or CAT [31]. TACR thus demonstrates that enhancing mitochondrial PDT does not necessarily involve increasing total oxygen production. An equally effective strategy may be to reduce the competitive consumption of O_2_ by the respiratory chain, thereby increasing its availability for the photodynamic reaction taking place in the immediate vicinity of the mitochondria.

### 4.3. Impaired Antioxidant Defence and Metabolic Adaptation in TNBC

A different strategy was employed in the MSPC, where an increase in oxidative stress was achieved by simultaneously impairing the antioxidant systems and disrupting the metabolic adaptation of cancer cells [23]. Glutathione (GSH) is one of the main intracellular systems maintaining redox homeostasis and is one of the most abundant cellular antioxidants [65]. It plays a role in the neutralisation of ROS both directly and as an electron donor for enzymatic antioxidant systems, in particular glutathione peroxidases [66]. The significance of this pathway in breast cancer is confirmed by studies of clinical samples, which have shown increased levels of GSH in tumour tissue compared with normal breast tissue [67]. The changes also affect enzymes involved in glutathione metabolism, although their expression and activity show considerable heterogeneity between individual tumours [68]. Upon release from the PEITC nanocarrier, it reduced GSH levels in 4T1 cells, limiting their ability to neutralise oxidative stress. At the same time, photoactivation of SQ-675 triggered the release of CO from CORM-401; in 4T1 cells, a distinct CO signal appeared primarily following irradiation with MSPC. CO disrupted mitochondrial respiration, and this effect overlapped with the mitochondrial damage induced by PDT. In the MSPC + light group, the inhibition of ATP production reached 76.31 per cent, whilst basal respiration was reduced by 45.08 per cent. Over time, a decrease in lactate levels was also observed, which the authors interpreted as a reduction in compensatory glycolysis following an initial increase in the activity of this pathway. MSPC therefore acts at two complementary levels of the cell’s response to oxidative stress. PEITC reduces GSH-dependent antioxidant capacity, whilst CO released from CORM-401 disrupts mitochondrial bioenergetics and impairs the cell’s ability to metabolically compensate for photodynamic damage to mitochondria [23]. Mitochondria are an important molecular target for CO; this gas can interact with the haem centre of cytochrome c oxidase (complex IV), leading to the inhibition of its activity and the modulation of mitochondrial respiration [69,70]. This effect depends on the dose, duration of exposure and oxygen conditions; therefore, CO should not be regarded solely as a simple inhibitor of mitochondrial respiration. In the case of CORM-401, the source of CO used in the MSPC, impaired oxidative coupling, a reduction in mitochondrial respiratory reserve and ATP turnover, and an effect on glycolysis have been demonstrated [71]. With prolonged exposure to CO released from CORM-401, inhibition of mitochondrial respiration and a reduction in mitochondrial ATP production were also observed [72]. Against this background, the MSPC mechanism can be interpreted as a simultaneous effect on two complementary components of the cell’s response to oxidative stress. PEITC reduces GSH-dependent antioxidant capacity, whilst CO released from CORM-401 disrupts mitochondrial bioenergetics and limits the cell’s ability to metabolically compensate for photodynamic damage to the mitochondria [23]. As a result, MSPC not only increases oxidative stress, but also impairs the cell’s ability to neutralise it and to adapt energetically following mitochondrial damage.

### 4.4. Photophysical and Photothermal Enhancement of Mitochondrial PDT

The optical properties of the nanocarrier may enhance the efficacy of mitochondrial PDT by modifying light absorption, ROS generation efficiency, or by combining the photodynamic effect with local hyperthermia [73,74]. In the studies analysed, these strategies were primarily utilised in systems based on gold nanorods and carbon-based materials. In the VO@AuNRs nanoconjugate, the coupling of the oxovanadium(IV) complex with gold nanorods modified the photophysical properties of the system: the maximum of the longitudinal plasmon resonance shifted from 712 to 845 nm, and the quantum yield for ^1O_2_ generation increased from 0.54 for free VO to 0.61 for VO@AuNRs. Importantly, this effect was reflected at the cellular level. In MDA-MB-231 cells, irradiated VO@AuNRs generated more intracellular ROS than the free VO complex and induced more pronounced depolarisation of the mitochondrial membrane. Since the functionalisation of AuNRs also increased cellular uptake and the mitochondrial localisation of the complex, the enhanced photodynamic effect cannot be attributed solely to increased ^1O_2_ generation; it likely resulted from the combined effects of improved photoreactivity, more efficient cellular delivery and preferential interaction with mitochondria [35]. A different mechanism was employed in CN/IR780, where porous carbon nanoparticles acted simultaneously as both an IR780 carrier and a photothermal component. Upon irradiation at 808 nm, the system achieved a photothermal conversion efficiency of 34.36 per cent and maintained stable heating capacity across successive cycles. In 4T1 cells, CN/IR780 + NIR induced the strongest increase in intracellular ROS and a marked loss of mitochondrial membrane potential, exceeding the effect of free IR780 and irradiated carbon nanoparticles alone. The authors partly attributed the increased ROS generation to the local photothermal effect; however, the experimental setup used did not allow for a clear distinction between the effect of hyperthermia on ROS photogenesis and the effects of increased IR780 delivery and direct thermal damage [27]. CN/IR780 should therefore be interpreted primarily as a platform for synergistic PDT/PTT, in which both mechanisms jointly exacerbate oxidative stress and mitochondrial dysfunction, rather than as unequivocal evidence of a thermal increase in the photosensitiser’s photochemical efficiency. Both systems demonstrate that the physical properties of the nanocarrier can enhance mitochondrial oxidative stress independently of the organelle-targeting mechanism itself. In VO@AuNRs, this effect stems mainly from modifications to the optical properties and increased generation of 1O_2_ [35], whereas in the CN/IR780, the combination of ROS generation and local hyperthermia [27]. However, any interpretation of the efficacy of such platforms must take into account the interplay between photophysics, cellular uptake, mitochondrial localisation and the direct thermal effect.

## 5. From Mitochondrial Photodamage to Regulated Cell Death and Antitumour Immunity

Targeted ROS production in mitochondria can lead to a loss of mitochondrial membrane potential, disruption of energy metabolism and the activation of regulated mechanisms of cell death [75,76]. However, the consequences of mitochondrial photodamage are not limited to direct cytotoxicity. Depending on the nature and severity of the stress, PDT may lead to the release of immunogenic signals and the initiation of innate and adaptive antitumour responses [77,78,79]. In the context of mitochondria-targeted phototherapy, the involvement of various forms of regulated cell death, including apoptosis and pyroptosis, has been described; in some systems, these were accompanied by an immunogenic cell death phenotype [75].

### 5.1. Mitochondrial Dysfunction and Apoptosis

The mitochondrial pathway of apoptosis is directly linked to the permeabilisation of the outer mitochondrial membrane (MOMP), which leads to the release of cytochrome c and other pro-apoptotic factors into the cytoplasm. Cytochrome c then participates in the formation of the apoptosome with APAF1, leading to the activation of caspase-9, followed by the effector caspases caspase-3 and caspase-7 [80,81]. In the studies analysed, however, the extent to which this mechanism was confirmed varied. A loss of mitochondrial membrane potential (ΔΨm) indicates mitochondrial dysfunction, but does not in itself confirm the activation of the intrinsic apoptotic pathway, for which MOMP, the release of cytochrome c and the subsequent activation of caspases are more direct indicators [82,83]. The most comprehensive evidence was presented for VO@AuNRs. Following irradiation of MDA-MB-231 cells, the nanoconjugate induced marked depolarisation of the mitochondrial membrane and 55.44% total cell death, comprising 1.42% of cells in early apoptosis, 50.23% in late apoptosis and 3.79% necrotic cells. For the free VO complex, the total percentage of dead cells was 31.01%. Immunoblotting simultaneously revealed an increase in the levels of Bax, cleaved caspase-3 and cleaved PARP, as well as a decrease in the expression of Bcl-2, Bcl-xL and survivin following treatment with VO@AuNRs and light [35]. Taken together, these results provide relatively strong evidence for the activation of mitochondria-associated intrinsic apoptosis. Mitochondrial dysfunction and apoptosis were also observed following the application of MSPC. Irradiation caused a marked reduction in ΔΨm in 4T1 cells, and Annexin V/PI analysis revealed the highest proportion of apoptotic cells following the application of MSPC in combination with light. This effect should be interpreted as the result of the cumulative action of mitochondrial PDT, GSH depletion and disruption of energy metabolism by CO [23]. In the TACR system, irradiation led to approximately 56% apoptosis in 4T1 cells, which was the highest result among the formulations compared. In the case of CN/IR780, Annexin V/PI analysis showed 34.87% apoptosis following treatment with the nanoplatform and NIR radiation, compared with 23.16% for carbon nanoparticles alone with NIR [31]. The accompanying depolarisation of the mitochondria indicates their involvement in the process of cell death [27], However, the lack of analysis of key components of the intrinsic apoptotic pathway, such as BAX/BAK activation, the release of cytochrome c or caspase-9 activation, means that the observed effect cannot be unequivocally attributed to classical mitochondrial apoptosis [81,82].

### 5.2. Pyroptosis and the Co-Occurrence of Various Mechanisms of Cell Death

Mitochondrial oxidative stress may be involved in the induction of pyroptosis, a lytic form of regulated cell death dependent on the activation of gasdermins and the formation of pores in the cell membrane by their N-terminal fragments [84]. Mitochondrial ROS may promote the activation of the NLRP3 inflammasome [85], and also directly increase the susceptibility of GSDMD to activation through oxidative modification of this protein, thereby intensifying pyroptotic cell death [86]. Of the systems analysed, the strongest evidence for this mechanism was found for MSPC. Following irradiation of 4T1 cells, increased levels of cleaved caspase-1 and the N-terminal fragment of GSDMD (GSDMD-N) were observed, indicating activation of the caspase-1/GSDMD axis. As increased apoptosis was also demonstrated under the same conditions, the results suggest the co-existence of several programmes of regulated cell death, rather than the exclusive activation of a single mechanism [23]. Apoptosis and pyroptosis have also been proposed as mechanisms of action for TTP-M. Following photoactivation, apoptotic features were observed in the Annexin V/PI assay, along with a reduction in the levels of full-length caspase-3 and GSDMD in HeLa cells, which the authors interpreted as a consequence of their proteolytic activation. Following prolonged incubation, allowing for the mitochondrial accumulation of TTP-M, similar changes were observed following irradiation. However, the interpretation of pyroptosis in this system requires greater caution than in the case of MSPC [24]. Direct detection of the GSDMD-N porin fragment provides stronger evidence of pyroptosis activation than a reduction in the level of full-length GSDMD alone, as the proteolytic release of the N-terminal domain is a key step leading to its translocation to the membrane and the formation of pores [87,88]. Furthermore, detailed mechanistic studies of TTP-M have been carried out mainly in HeLa cells; consequently, their direct extrapolation to TNBC remains limited [24].

### 5.3. Immunogenic Cell Death as a Link Between Photodamage and the Immune Response

ICD does not constitute a distinct mechanism of cell death parallel to apoptosis or pyroptosis, but rather defines the functionally immunogenic nature of regulated cell death, capable of initiating a specific adaptive immune response [77,89]. The canonical signals associated with ICD include the translocation of calreticulin (CRT) to the cell surface and the release of ATP and HMGB1 [90]. These signals promote the recruitment, phagocytosis, maturation and activation of antigen-presenting cells, particularly dendritic cells, as well as the presentation of tumour antigens to T lymphocytes, thereby laying the foundation for the development of an adaptive antitumour response [77,90]. A particularly clear link between mitochondrial oxidative stress and ICD was demonstrated for Rh-PTZ. In 4T1 cells, mitochondrial PDT increased the surface exposure of CRT, the release of HMGB1 from the nucleus, and the concentration of extracellular ATP. This was accompanied by an increase in the secretion of TNF-α and IL-6. In the 4T1 model, treatment with Rh-PTZ combined with irradiation increased the maturation of dendritic cells within the tumour and the infiltration of CD8+ lymphocytes; the proportion of CD8+ T cells reached 20.4 per cent. At the same time, the proportion of immunosuppressive MDSCs decreased to 26.1 per cent [22]. These results suggest that mitochondrial photooxidation may lead from local damage to the organelle to an immunological remodelling of the tumour microenvironment. A similar effect was observed for TACR. Following irradiation of 4T1 cells, the intensity of the surface CRT signal was approximately three times greater than that for TACR without light, whilst the release of HMGB1 and ATP was also observed. The supernatant from such treated cells induced the maturation of 48.1% of BMDCs with the CD11c+CD80+MHCII+ phenotype [31]. These results directly link the increase in photodynamic mitochondrial damage following improvement in oxygen balance to the development of an immunogenic cell death phenotype. HABH also induced CRT exposure and the release of HMGB1 and ATP by 4T1 cells. The supernatant following treatment with HABH and light increased the proportion of mature BMDCs to 20.52 per cent, whilst the proportion of M1-like macrophages (F4/80+CD11c+) reached 31.81 per cent [39]. In this system, however, ICD was only one component of the immune response, as mitochondrial damage was also associated with the activation of the cGAS–STING axis. It should be emphasised that the canonical ICD hallmarks discussed above, including CRT exposure and extracellular release of ATP and HMGB1, were assessed predominantly in vitro. In vivo experiments mainly demonstrated downstream immune consequences, such as dendritic-cell maturation, cytotoxic T-cell infiltration and systemic antitumour responses, rather than direct confirmation of ICD within tumour tissue. Therefore, the available in vivo immune data support the immunological relevance of PDT-induced cell death but should not be regarded as definitive evidence of ICD in vivo across all included studies.

### 5.4. Cytoplasmic DNA Accumulation and Activation of the cGAS–STING Axis

The release of mitochondrial DNA (mtDNA) into the cytosol can lead to its recognition by cGAS, the synthesis of cGAMP and the activation of the STING–TBK1–IRF3 pathway, resulting in the induction of type I interferons [91,92]. This mechanism represents a direct molecular link between mitochondrial damage and the activation of the innate immune response [91,92,93]. This relationship was utilised most comprehensively in the HABH system. Following photoactivation in 4T1 cells, a 29.30 per cent increase in cytoplasmic dsDNA was observed compared with the control. At the same time, levels of cGAMP and STING, as well as the phosphorylation of TBK1 and IRF3, increased. IFN-β secretion was approximately 2.6 times higher than in the control group. These results provide convincing evidence of cGAS–STING pathway activation following photodynamic damage to cells. However, the claim that this pathway is unequivocally mtDNA-dependent should be interpreted with caution. The authors demonstrated an increase in cytoplasmic dsDNA and linked this to mitochondrial photodamage; however, the quantitative assay used did not directly distinguish between mtDNA and nuclear DNA. The concurrent increase in the DNA damage marker γ-H2AX indicates that nuclear material was also damaged [39]. Accordingly, although mitochondrial photodamage provides a plausible source of cytosolic DNA, a contribution from nuclear DNA-derived fragments cannot be excluded on the basis of the available data. Therefore, the most accurate interpretation is that HABH induced mitochondrial stress and cytoplasmic DNA accumulation, leading to the activation of cGAS–STING, whilst a predominant contribution from mtDNA would require the direct quantification of mitochondrial sequences in the cytoplasmic fraction.

### 5.5. From Local ICD to Systemic Antitumour Immunity

One of the most significant functional consequences of ICD and the activation of innate immune pathways is the ability to transform the local effect of PDT into a systemic antitumour response, capable of limiting the growth of lesions located outside the immediate field of irradiation [94]. This has been most thoroughly investigated for TACR and HABH. In the bilateral 4T1 model, TACR combined with irradiation inhibited the growth of both the primary tumour and the non-irradiated distant tumour. The combination of TACR-mediated PDT with the α-PD-L1 antibody further enhanced the inhibition of both tumours. The proportion of mature DCs in the tumour-draining lymph nodes was 44.26% following TACR + light and 49.46% following TACR + light + α-PD-L1. In the primary tumour, the proportion of CD8+ T cells increased from 1.99% in the PBS group to 5.92% following TACR + light and 7.36% following combination therapy. In the distant tumour, the corresponding values were 1.92%, 6.95% and 4.69% [31]. antitumour These findings support the induction of systemic immune-associated antitumour effects following TACR-mediated PDT; however, they do not by themselves demonstrate durable systemic antitumour immunity. The benefit of PD-L1 blockade is best demonstrated in terms of tumour growth inhibition, rather than through a uniform increase in each of the immunological parameters analysed. HABH also elicited a marked immune response in vivo. In the 4T1 orthotopic model, the concentration of IFN-β following HABH + light was approximately 4.6 times higher than in the control. The proportion of mature DCs in tumour tissue increased from 8.34% to 20.27%, whilst the infiltration of CD3+CD8+ T cells rose from 4.03% to 11.36%. Concurrently, an increase in the populations of M1-like macrophages, mature DCs and cytotoxic T cells was observed in the lymph nodes. Importantly, mice in which prior treatment with HABH + light had led to tumour elimination showed limited growth of re-implanted 4T1 cells without further administration of the therapy [39], which indicates the development of a sustained antitumour immune response.

Overall, the immunological evidence across the included studies remains predominantly preclinical and should be interpreted cautiously. Increased CRT exposure, HMGB1 and ATP release, dendritic-cell maturation, cytokine production and immune-cell infiltration are consistent with immune activation, but these endpoints are largely surrogate or correlative and do not by themselves demonstrate durable systemic antitumour immunity. More compelling functional evidence was provided by distant-tumour control in the TACR study [31] and by tumour rechallenge in the HABH study [39]; however, such experiments were not performed consistently across the included platforms. Consequently, durable systemic immunity cannot currently be regarded as a general feature of mitochondria-targeted nanoparticle PDT in TNBC.

## 6. Preclinical Evidence in TNBC Models

The available preclinical evidence suggests antitumour activity of mitochondria-targeted PDT in selected models; however, the range of models used remains relatively limited. Most of the studies analysed utilised 4T1 mouse cells and syngeneic models in immunocompetent BALB/c mice, with the exception of MSPC, which was evaluated in vivo in immunodeficient BALB/c nude mice; amongst human TNBC models, the MDA-MB-231 cell line was predominant. The 4T1/BALB/c model is a well-characterised syngeneic TNBC model which preserves the host’s functional immune system, enabling the simultaneous assessment of direct anticancer activity and the therapy-induced immune response [95,96]. However, the biological characteristics of 4T1 should also be considered when interpreting therapeutic responses. This model is highly aggressive and metastatic and is associated with a strongly immunosuppressive, myeloid-rich tumour microenvironment, which may not reproduce the immune architecture of all human TNBCs [97]. In addition, 4T1 tumours display distinctive metabolic features, including high glycolytic activity, pronounced hypoxia and extracellular acidosis, as well as substantial intratumoural metabolic heterogeneity [98]. These properties may directly influence PDT response because oxygen availability, redox balance and mitochondrial metabolism are central determinants of photodynamic efficacy. Consequently, therapeutic and immunological effects observed in 4T1 models should not be assumed to generalise directly to the broader spectrum of human TNBC. MDA-MB-231, on the other hand, is a widely used human TNBC cell line with a mesenchymal phenotype; however, a single cell line does not reflect the full molecular and immunological heterogeneity of TNBC [99,100]. The predominance of the 4T1 and MDA-MB-231 models therefore limits the ability to assess the efficacy of these strategies across a broader spectrum of biological subtypes of human TNBC.

### 6.1. In Vitro Evidence

All the nanoplatforms analysed exhibited light-dependent cytotoxic activity in TNBC models, with relatively little effect under non-irradiated conditions. This effect was observed both in human MDA-MB-231 cells for VO@AuNRs and in mouse 4T1 cells for Rh-PTZ, MSPC, HABH, TACR, TTP-M NPs and CN/IR780 [22,23,24,27,31,35,39]. Importantly, the effects of these systems were not limited to reducing cell lifespan. Depending on the platform, increased ROS production, loss of mitochondrial membrane potential, bioenergetic disturbances and the activation of regulated cell death mechanisms were also observed [22,23,27,31,35]. These results indicate that the observed photocytotoxicity was associated with direct impairment of mitochondrial function, although the scope of the mechanistic analyses varied between individual studies. It is worth noting, however, that not all detailed mechanistic experiments were carried out directly in TNBC. In the case of TTP-M, some of the analyses concerning sequential subcellular localisation and mechanisms of cell death were primarily conducted in HeLa cells, whilst the anticancer activity of the nanoparticles was also confirmed in 4T1 cells [24]. This limits the extent to which the specific mechanisms of this system can be directly extrapolated to TNBC.

### 6.2. In Vivo Efficacy

The most direct evidence of the efficacy of mitochondrial PDT was obtained in syngeneic 4T1 models. In the subcutaneous 4T1 model, the use of Rh-PTZ in combination with irradiation led, after 14 days, to a reduction in the mean tumour weight to 5.4 per cent of the value observed in the PBS group. Histological examination confirmed extensive damage to tumour tissue and an increased number of TUNEL-positive cells [22]. MSPC was evaluated in the 4T1-Luc model in BALB/c nude mice. The combination of the nanoplatform with irradiation resulted in a 75.44% inhibition of tumour growth, whilst the inhibition index calculated on the basis of the final tumour weight was 75.52%. The tumour weight following MSPC + light treatment was 24.48% of that observed in the PBS + light control group [23]. In the subcutaneous 4T1 model, intravenous administration of TTP-M NPs in combination with irradiation reduced the tumour volume to approximately 11 mm^3^, and the mean tumour weight was 0.072 g; the authors calculated a 65.1% inhibition of tumour growth. By comparison, in the control group, after 8 days, the tumour volume was approximately 240 mm^3^ and its mass was approximately 0.45 g [24]. HABH exhibited strong activity in the 4T1 orthotopic model. Following two administrations of the system and photoactivation, the strongest tumour growth inhibition was observed amongst the groups analysed. In an extended 16-day experiment, HABH + light therapy led to the almost complete elimination of tumour tissue. Importantly, re-implantation of 4T1 cells into mice in which tumour elimination had previously been achieved led to a marked reduction in the growth of the subsequent tumour without the need for repeat administration of the therapy, providing functional evidence of a sustained protective antitumour response after tumour rechallenge [39]. TACR was evaluated in both unilateral and bilateral 4T1 models. TACR + light inhibited the growth of the primary tumour and, in the bilateral model, also limited the growth of the non-irradiated distant tumour. The addition of an anti-PD-L1 antibody further enhanced control of both tumour lesions, providing functional evidence of the combination of local PDT with a systemic antitumour response. [31]. In the subcutaneous 4T1 model, CN/IR780, when combined with 808 nm radiation, resulted in the strongest inhibition of tumour growth amongst the groups assessed, and the authors described the almost complete disappearance of the tumour following treatment with CN/IR780 + NIR [27]. It should be borne in mind, however, that this effect resulted from the combined action of PDT and PTT, and therefore cannot be attributed solely to mitochondrial phototoxicity. VO@AuNRs, on the other hand, represents an important exception: despite extensive studies in human MDA-MB-231 cells, covering photocytotoxicity, apoptosis, migration and EMT markers, no in vivo antitumour validation was carried out in this study [35]. Conclusions regarding its therapeutic potential therefore remain at the level of cell-based research.

### 6.3. Distribution and Pre-Clinical Safety

In most studies, short-term tolerance of the nanoplatforms was favourable. Rh-PTZ did not cause any significant changes in body weight, liver or kidney function parameters, or histopathological changes in the major organs [22]. Following MSPC + light, no significant differences in body weight or histological changes in the organs were observed; furthermore, rapid renal clearance of the ultrasmall nanodots was demonstrated, accompanied by a prolonged signal within the tumour [23]. TTP-M NPs showed haemolysis of <0.1 per cent, no significant changes in ALT, AST and BUN, and no detectable histopathological changes in the major organs [24]. Favourable results from short-term histological and/or haematological assessments were also reported for HABH, TACR and CN/IR780 [27,31,39]. These data indicate favourable short-term preclinical tolerability of the nanoplatforms under investigation; however, they are not equivalent to a comprehensive toxicological assessment, which should take into account, amongst other things, the biodistribution and fate of the nanomaterial in vivo, toxicity following repeated administration, immunotoxicity and the potential effects of long-term exposure [101]. Most studies involved small groups of animals and an observation period of a few to a dozen or so days, whilst the long-term retention of inorganic materials, immunotoxicity, the pharmacokinetics of individual components, and toxicity following repeated administration remain poorly characterised. Overall, the available preclinical studies suggest that mitochondrial targeting may enhance the efficacy of nanoparticle-mediated PDT in selected TNBC models; however, the evidence remains preliminary and does not yet allow this effect to be generalised across different nanoplatforms or TNBC subtypes. However, the strength of the evidence is uneven: the most advanced studies involve immunocompetent 4T1 models and demonstrate control of the primary tumour, an effect on distant lesions, or a sustained protective antitumour response after tumour rechallenge [22,31,39], whilst validation in human TNBC models, PDX models and spontaneous metastasis models remains limited. Future studies should therefore extend validation beyond 4T1-based systems and incorporate multiple human TNBC models representing distinct molecular and metabolic phenotypes. In particular, patient-derived xenografts and patient-derived three-dimensional models could better preserve intertumoural heterogeneity, while orthotopic and spontaneous or experimental metastasis models would allow evaluation of therapeutic efficacy in clinically relevant patterns of tumour dissemination. For studies focused on immune activation, immunocompetent models remain essential, whereas complementary human-derived models are needed to determine whether the mitochondrial vulnerabilities identified in murine 4T1 cells are conserved across human TNBC. A combined model strategy would therefore provide a more rigorous basis for assessing both therapeutic efficacy and translational relevance (Table 1).

### 6.4. Comparative Synthesis of Mitochondrial Targeting and Therapeutic Performance

Cross-study comparison reveals substantial differences in both the strength of mitochondrial-targeting evidence and the reported therapeutic outcomes. With respect to mitochondrial localisation, reported PCC values varied considerably among the included studies: approximately 0.97 for TTP-M, predominantly in HeLa cells [24], 0.94 for VO@AuNRs in MDA-MB-231 cells [35], 0.92 for HABH-delivered BDP in 4T1 cells [39], 0.91 for Rh-PTZ in 4T1 cells [22], approximately 0.84–0.88 for MSPC/SQ-675 [23], and 0.54 for TACR compared with 0.36 for the non-TPP control [31]. CN/IR780 lacked direct mitochondrial colocalisation analysis [27]. Importantly, these values are reported here descriptively and should not be interpreted as a cross-platform ranking of mitochondrial-targeting efficiency [51,52,53,54].

Therapeutic efficacy was likewise difficult to compare directly because different studies used distinct photosensitiser doses, irradiation wavelengths, light doses, treatment schedules, tumour models and efficacy endpoints. Nevertheless, substantial tumour control was reported across several 4T1 models: Rh-PTZ reduced final tumour mass to 5.4% of the PBS control [22], MSPC achieved a tumour growth inhibition of 75.44% [23], and TTP-M NPs produced a reported tumour growth inhibition of 65.1% [24]. HABH produced near-complete tumour suppression in an extended 4T1 experiment [39], while TACR additionally inhibited non-irradiated distant tumours and benefited from combination with anti-PD-L1 therapy [31]. CN/IR780 also produced marked tumour suppression, although its therapeutic effect reflected combined PDT/PTT and therefore cannot be attributed solely to mitochondrial PDT [27]. VO@AuNRs was evaluated only in vitro in TNBC models [35]. Because of these methodological differences, the available efficacy data support proof-of-concept activity but do not permit a reliable ranking of the platforms.

The evidence for immune activation was even more heterogeneous. Rh-PTZ, TACR and HABH showed canonical ICD-associated markers, including CRT exposure and ATP/HMGB1 release, together with downstream changes in dendritic cells or T-cell infiltration [22,31,39]. TACR provided additional functional evidence through inhibition of a distant non-irradiated tumour [31], whereas HABH showed reduced growth after tumour rechallenge [39]. However, most immunological endpoints were surrogate or correlative measurements, and their experimental designs differed substantially. Thus, the available data suggest that mitochondrial photodamage can contribute to antitumour immune activation, but they do not establish that all mitochondria-targeted platforms produce comparable or durable systemic immunity.

A further distinction emerges between platform complexity and demonstrated therapeutic benefit. Relatively simple systems such as F127/Rh-PTZ rely primarily on the intrinsic mitochondrial affinity and photochemistry of the photosensitiser [22], whereas multicomponent platforms such as MSPC, TACR and HABH additionally incorporate metabolic regulation, oxygen modulation, catalytic activity or immune-related functions [23,31,39]. These more complex systems often produced broader biological effects, but the available studies rarely included direct head-to-head comparisons with simpler mitochondria-targeted or non-targeted nanoparticle PDT formulations under equivalent experimental conditions. Consequently, it remains unclear whether the additional therapeutic effects are sufficiently large and reproducible to justify the increased formulation complexity.

## 7. Translational Challenges and Future Directions

The clinical necessity of mitochondrial targeting in nanoparticle-mediated PDT has not yet been established. Its biological rationale is compelling because the short diffusion range of photogenerated ROS makes subcellular localisation an important determinant of photodamage, while organelle-targeted phototherapy has been proposed as a means of increasing the spatial precision and therapeutic efficiency of PDT [102,103]. However, a mechanistic rationale alone is insufficient to justify additional nanoplatform complexity. From a translational perspective, increasing structural and functional complexity introduces additional challenges related to physicochemical characterisation, reproducibility, manufacturing, scale-up and regulatory development [104]. To provide a clinically meaningful advantage, mitochondrial targeting should therefore demonstrably improve outcomes such as tumour control, therapeutic index, effective photosensitiser or light dose, activity under treatment-resistant conditions, or induction of therapeutically relevant immune responses compared with simpler nanoparticle PDT systems.

Among the studies included in this review, direct evidence isolating the specific contribution of mitochondrial targeting remains limited. TACR provides one of the clearest comparative designs because a corresponding formulation lacking the TPP mitochondrial-targeting moiety was used as a control [31]. In most other studies, mitochondrial targeting was introduced together with changes in cellular uptake, photosensitiser delivery, oxygen regulation, redox modulation, catalytic activity or photothermal effects [22,23,24,27,35,39], making it difficult to attribute improved therapeutic efficacy specifically to mitochondrial localisation. Therefore, future studies should include matched mitochondria-targeted and non-targeted formulations with otherwise equivalent physicochemical properties, alongside simpler PDT formulations and clinically relevant comparators. Only such studies can determine whether the additional therapeutic benefit is sufficiently large and reproducible to justify the increased formulation complexity and translational burden.

One of the most significant limitations inherent to PDT itself remains the controlled delivery of an adequate dose of light to the entire tumour volume. The therapeutic effect depends not only on the light energy delivered, but also on the local concentration of the photosensitiser, the availability of oxygen, and the spatial distribution of fluence and fluence rate within the tissue [105,106]. For this reason, the fluence delivered to the surface does not directly reflect the actual photodynamic dose within the tumour, as the propagation of light is further modulated by absorption and scattering dependent on the optical properties of the tissue [105]. The introduction of dosimetry that takes into account the spatial distribution of light, PS concentration and local oxidation will be particularly important when moving on to larger and more heterogeneous tumour volumes of clinical size [106]. In the case of deep-seated lesions or those of considerable thickness, it may be necessary to deliver light interstitially via optical fibres, enabling more controlled irradiation of the entire tumour volume [106,107].

Another significant translational barrier is the ability to reproducibly manufacture complex nanoplatforms on a clinical scale. Multi-step synthesis processes, the co-encapsulation of several active substances and complex surface modifications increase the number of critical quality attributes and process parameters that require control between batches [101,108]. This issue is particularly significant in the case of platforms containing biological components, where variability at source and multi-step isolation and functionalisation procedures further complicate standardisation and the assurance of reproducibility [109]. Scaling up the process may lead to changes in particle size and size distribution, surface charge and cargo loading, thereby affecting the biological properties and delivery efficiency [101,110]. For this reason, clinical development should favour designs that are as simple as possible whilst retaining the functions necessary to achieve a therapeutic advantage, and should incorporate the principles of quality-by-design (QbD) from the early stages of formulation and manufacturing process development [108,111].

An important area for further research will be the identification of patients most susceptible to mitochondria-targeted PDT. TNBC is a biologically and molecularly heterogeneous group of tumours, comprising subtypes with different molecular and immunological profiles and potential therapeutic susceptibilities [99]. This heterogeneity also extends to tumour metabolism and mitochondrial function; in TNBC, distinct metabolic profiles have been described, including, amongst other things, differences in the utilisation of glycolysis, lipid metabolism and oxidative phosphorylation, which may translate into varying susceptibility to metabolically targeted strategies [112,113]. It can therefore be assumed that the efficacy of strategies utilising the potential of the mitochondrial membrane, hypoxia, oxidative metabolism or specific antioxidant systems will not be uniform across all TNBCs. Candidate biomarkers, including the tumour’s bioenergetic and redox profile, the degree of hypoxia, and the expression of receptors used for the first stage of targeting, should be evaluated prospectively in relation to therapeutic response. This approach is consistent with the broader trend in the development of oncological nanomedicine, in which patient stratification using biomarkers and companion diagnostics is recognised as a key element in improving the translation of nanomedicine systems [114].

It will also be necessary to define the clinical role of mitochondrial nano-PDT. Its potential value may extend beyond local cytoreduction to include the use of controlled photodynamic stress to induce immunogenic cell death and increase tumour susceptibility to immunotherapy [115,116]. From this perspective, PDT optimisation should not focus solely on maximising direct cytotoxicity, but should also take into account the irradiation parameters and treatment regimen that influence the nature of the immune response [106]. Future research should therefore combine the simplification of nanoplatform design, the standardisation of dosimetry, and the prospective identification of biomarkers that can determine in which patients and in what clinical context mitochondrial targeting actually offers an advantage over conventional PDT.

## 8. Conclusions

Current evidence suggests that mitochondria-targeted nanoparticle PDT may offer therapeutic benefits in selected preclinical TNBC models by combining localised ROS generation with disruption of mitochondrial bioenergetics, redox homeostasis and cell-death pathways. However, this evidence is based on only seven highly heterogeneous studies and should therefore be considered preliminary. It remains unclear whether mitochondrial targeting itself provides a consistent advantage over simpler nanoparticle PDT systems. Further validation in diverse and clinically representative TNBC models, together with direct comparative studies, will be required before broader conclusions regarding therapeutic superiority or translational potential can be drawn.

## Figures and Tables

**Table 1:** Experimental strength of evidence for mitochondrial targeting and preclinical efficacy of nanoparticle-mediated PDT in TNBC.

Nanoplatform	TNBC Model	Evidence for Mitochondrial Localisation and Tracked Component	Strength of Evidence	Main Amplification Strategy	Key Biological Outcome	In Vivo Evidence	Main Limitation
Rh-PTZ/F127	4T1	Direct colocalisation with MitoTracker; PCC = 0.91	Direct PS-level evidence in TNBC cells	Type I PDT; radical ROS generation	Mitochondrial dysfunction, apoptosis, ICD	Subcutaneous 4T1; final tumour mass 5.4% of PBS control	Localisation reflects Rh-PTZ signal, not intact F127 nanoparticles
MSPC	4T1/4T1-Luc	Time-dependent colocalisation of SQ-675; PCC 0.58 at 2 h, 0.82 at 4 h, ~0.84–0.88 at 6 h	Direct PS-level evidence in TNBC cells	GSH depletion, CO-mediated respiratory interference, metabolic disruption	Apoptosis and pyroptosis	4T1-Luc in BALB/c nude mice; TGI 75.44%	In vivo model was immunodeficient; mitochondrial localisation mainly reflects released SQ-675
TTP-M NPs	4T1; mechanistic studies mainly HeLa	PCC ≈ 0.97; detailed localisation analysis mainly performed in HeLa cell	Direct evidence, but mainly outside TNBC models	Type I/II ROS; sequential membrane-to-mitochondria redistribution	Apoptosis and proposed pyroptosis	Subcutaneous 4T1; tumour inhibition 65.1%	Mitochondrial mechanism not validated with comparable detail directly in TNBC cells
CN/IR780	4T1	Indirect evidence; mitochondrial dysfunction observed, but no direct organelle colocalisation	Indirect evidence	Combined PDT/PTT	ROS generation, ΔΨm loss, apoptosis	Subcutaneous 4T1; marked tumour suppression	Mitochondrial targeting not directly demonstrated; efficacy reflects combined PDT/PTT
TACR	4T1	PCC = 0.54 vs. 0.36 for formulation without TPP	Direct PS-level evidence with non-targeted control	CAT-mediated O_2_ generation + atovaquone-mediated reduction in O_2_ consumption	Apoptosis, ICD, systemic immune-associated antitumour effects tumour	Single and bilateral 4T1 models; enhanced by anti-PD-L1	Moderate PCC; localisation mainly reflects TPP-Ppa rather than the complete nanoplatform
HABH	4T1	Direct BDP mitochondrial colocalisation; PCC = 0.92, OLC = 0.93	Direct PS-level evidence in TNBC cells	Type I PDT + nanozyme-mediated •OH generation	ICD, cGAS–STING activation, antitumour immunity	Orthotopic 4T1; strong tumour control and protective response after rechallenge	BDP also showed appreciable ER colocalisation; mitochondrial localisation was preferential, not exclusive
VO@AuNRs	MDA-MB-231	PCC = 0.94 vs. 0.63 for free VO	Direct quantitative colocalisation, but uptake-dependent contribution cannot be excluded	Plasmonic enhancement of ROS generation	Mitochondrial dysfunction and apoptosis	Not evaluated in a TNBC in vivo model	Increased mitochondrial colocalisation may partly reflect enhanced cellular uptake

## Data Availability

No new data were created or analysed in this study.
